# Risk of intellectual disability in children born appropriate-for-gestational-age at term or post-term: impact of birth weight for gestational age and gestational age

**DOI:** 10.1007/s10654-019-00590-7

**Published:** 2019-12-02

**Authors:** Ruoqing Chen, Kristina Tedroff, Eduardo Villamor, Donghao Lu, Sven Cnattingius

**Affiliations:** 1 Clinical Epidemiology Division, Department of Medicine Solna, Karolinska Institutet, 171 76 Stockholm, Sweden; 2 Department of Women’s and Children’s Health, Karolinska Institutet, Stockholm, Sweden; 3 Department of Epidemiology, School of Public Health, University of Michigan, Ann Arbor, MI USA; 4 Department of Medical Epidemiology and Biostatistics, Karolinska Institutet, Stockholm, Sweden; 5 Channing Division of Network Medicine, Brigham and Women’s Hospital and Harvard Medical School, Boston, MA USA; 6 Department of Epidemiology, Harvard T.H. Chan School of Public Health, Boston, MA USA

**Keywords:** Intellectual disability, Birth weight for gestational age, Gestational age, Cohort studies, Siblings

## Abstract

Children born small for gestational age have a higher risk of intellectual disability. We investigated associations of birth weight for gestational age percentile and gestational age with risk of intellectual disability in appropriate-for-gestational-age (AGA) children. We included 828,948 non-malformed term or post-term AGA singleton children (including 429,379 full siblings) born between 1998 and 2009 based on data from the Swedish Medical Birth Register. Diagnosis of intellectual disability after 3 years of age was identified through the Patient Register. Using Cox regression models, we calculated hazard ratios (HRs) with 95% confidence intervals (CIs) of intellectual disability among children with different birth weight percentiles and gestational age in the whole population and in a subpopulation of full siblings. A total of 1688 children were diagnosed with intellectual disability during follow-up. HRs (95% CIs) of intellectual disability for the low birth weight percentile groups (10th–24th and 25th–39th percentiles, respectively) versus the reference group (40th–59th percentiles) were 1.43 (1.22–1.67) and 1.28 (1.10–1.50) in population analysis and 1.52 (1.00–2.31) and 1.44 (1.00–2.09) in sibling comparison analysis. The increased risk for low birth weight percentiles in population analysis was stable irrespective of gestational age. A weak U-shaped association between gestational age and intellectual disability was observed in population analysis, although not in sibling comparison analysis. These findings suggest that among AGA children born at term or post-term, lower birth weight percentiles within the normal range are associated with increased risk of intellectual disability, regardless of gestational age.

**Electronic supplementary material:**

The online version of this article (10.1007/s10654-019-00590-7) contains supplementary material, which is available to authorized users.

## Introduction

Intellectual disability refers to a group of disorders characterized by significant cognitive limitations and limitation of adaptive functions that affects between 1 and 3% of the world’s population [1, 2]. Intellectual disability affects different aspects of life, including learning abilities, daily communication, self-care, social activities, etc. [1]. Fetal growth restriction may affect brain development and impair brain maturation and cognitive function [3]. Small for gestational age (SGA), defined as birth weight for gestational age below the population’s 10th percentile, has been associated with lower IQ and intellectual disability [4, 5]. However, SGA is not identical to fetal growth restriction: some SGA infants are constitutionally small and have reached their growth potential, whereas larger infants, e.g., infants with appropriate birth weight for gestational age (AGA, i.e., birth weight for gestational age between 10th and 90th percentiles), may have not fulfilled their biological growth potential—the weight a fetus ought to achieve in the absence of pathological conditions [6–8]. Low birth weight percentiles within the range of AGA have been associated with higher risk of neonatal neurological morbidity, such as convulsion and hypoxic ischemic encephalopathy, but rarely been investigated for long-term neurological outcomes [5, 9]. The association between birth weight for gestational age percentile (hereinafter called birth weight percentile) and risk of intellectual disability in AGA children has, to the best of our knowledge, not been specifically investigated in a nationwide population-based setting.

Preterm birth (< 37 gestational weeks) is another known risk factor for cognitive deficits [10]. The risk of intellectual disability increases exponentially as gestational age decreases in preterm children [11, 12]. A similar trend of increasing risk by decreasing gestational age was recently shown in children born at term (37–41 weeks), whereas a trend of increasing risk by increasing gestational age was observed in children born post-term (≥ 42 weeks) [12]. The same study also suggested a joint impact of term or post-term birth and SGA versus term birth and AGA. However, they did not explore the joint impact of birth weight percentile and gestational age within the AGA group.

Based on data from Swedish national registries, we aimed to investigate associations of birth weight percentile and gestational age with risk of intellectual disability in non-malformed, term or post-term, AGA children. We performed population analysis and sibling comparison analysis, the latter of which was to control for unmeasured familial confounding factors shared by siblings. We also assessed the joint impact of birth weight percentile and gestational age with regard to risk of intellectual disability.

## Methods

### Data sources

This was a cohort study based on data from several Swedish national registries, including the Medical Birth Register, Patient Register, Cause of Death Register, Education Register, Total Population Register, and Multi-Generation Register. The unique personal identity number assigned to all Swedish residents enables individual record linkage between different registries [13]. The Medical Birth Register includes standardized antenatal, obstetric and neonatal information for almost all deliveries in Sweden [14]. Information is collected from the first visit to antenatal care and throughout pregnancy, delivery, and the neonatal period. The Patient Register includes nationwide information on hospital discharge diagnoses from 1987, and diagnostic information on hospital-based outpatient visits from 2001 onward [15]. The Cause of Death Register includes information on dates and causes of death [16]. The Education Register, updated yearly, includes information about highest level of formal education [17]. The Total Population Register provides information on individuals’ demographic characteristics [18]. The Multi-Generation Register contains information on personal identity numbers of all first-degree relatives (i.e., parents, children, and siblings) of all residents in Sweden, which allows one to identify fathers and full siblings [19].

### Study participants

We identified all singleton live born infants between January 1st, 1998 and December 31st, 2009 from the Medical Birth Register (n = 1,136,671). We excluded infants who had missing personal identity numbers (n = 13,980), infants whose mothers (n = 426) or fathers (n = 7724) had missing personal identity numbers, infants with missing data on gestational age (n = 872), and infants with missing (n = 3198) or implausible (n = 702) data on birth weight for gestational age, the latter of which were defined as values below or above the 5 times standard deviation from the mean. We restricted the study population to infants born AGA at term or post-term, and, as a result, we excluded 54,288 preterm infants, 85,721 SGA infants and 103,202 large for gestational age (LGA) infants (i.e., birth weight for gestational age above the 90th percentile). We also excluded infants who were diagnosed with major malformations (including congenital malformations, deformations, and chromosomal abnormalities) as recorded in the Medical Birth Register or in the Patient Register during the first year of age (n = 30,077) (Supplementary Table 1 for the Swedish version of International Classification of Diseases, tenth revision [ICD-10] codes for major malformations).

Since reliable and validated assessment tools of intellectual function such as the Wechsler scale measurements, utilized in Sweden and globally, are only partially available from 2 years and 6 months of age [20], children were followed from their third birthday until the date of first diagnosis of intellectual disability, date of death, date of emigration, or December 31st, 2012, whichever came first. As a result, children who died (n = 810) or emigrated (n = 6723) before 3 years of age were excluded, leaving 828,948 children in the final analysis. Among the 828,948 children, 429,379 (51.8%) were full siblings.

### Exposure assessment

Birth weight was recorded for infants immediately after birth. Gestational age was assessed by ultrasonography offered during the early second trimester for 87.4% of all births, by the last menstrual period for 7.5% of all births, and by a postnatal assessment for 5.2% of all births in the cohort. From the ultrasound-based, sex-specific Swedish reference curve for fetal growth [21], we calculated the Z scores of birth weight for gestational age, which were further converted to birth weight percentiles.

Birth weight percentiles were categorized into five groups: 10th–24th, 25th–39th, 40th–59th (reference), 60th–74th, and 75th–90th percentiles [9]. Gestational age was categorized into five groups: 37–38 weeks, 39 weeks, 40 weeks (reference), 41 weeks, and ≥ 42 weeks. Birth weight percentiles and gestational age were also analyzed as continuous variables.

### Outcome ascertainment

Intellectual disability was defined as a hospital contact (either hospitalization or outpatient visit) with a clinical diagnosis of ICD-10 codes F70-F79 from the Patient Register. The severity of intellectual disability was further classified using corresponding ICD-10 codes (Supplementary Table 1). In a sensitivity analysis, we redefined the outcome as at least two hospital contacts for intellectual disability on separate dates. Information about clinical diagnosis and dates of admission and discharge were extracted from the Patient Register.

### Covariates

We identified maternal and neonatal variables that have been associated with both birth weight for gestational age/gestational age and risk of intellectual disability and might therefore confound the associations under study. As a result, we extracted information on maternal age at delivery [22, 23], parity [22, 24], smoking during pregnancy [7, 25], maternal height and weight collected at the first antenatal care visit, onset of labor [25, 26], mode of delivery [25, 27, 28], and child’s sex [22, 29] from the Medical Birth Register. Body mass index (BMI) in early pregnancy [22, 30] was calculated by dividing measured weight (kg) by self-reported height squared (m^2^). We also included information on calendar period of delivery from the Medical Birth Register to control for temporal change in obstetric practice. We further obtained information about maternal educational level [31, 32] from the Education Register and country of birth [33, 34] from the Total Population Register. Maternal diabetic and hypertensive diseases [7, 25] were defined by a diagnosis of the corresponding ICD-10 codes registered in the Medical Birth Register (Supplementary Table 1 for ICD-10 codes). All covariates were analyzed as categorical variables (Table 1 for categorization of covariates).

**Table 1:** Maternal and neonatal characteristics and rate of any intellectual disability in term or post-term, non-malformed, appropriate-for-gestational-age children (N = 828,948)

Characteristics	No. of children (N[%])	Intellectual disability	
		No. of cases	Rate (95%CI)^a^
Total	828,948	1688	3.59 (3.42–3.77)
Mothers			
Age at child’s birth (years)			
< 20	13,964 (1.7)	35	4.28 (3.08–5.97)
20–24	107,263 (12.9)	267	4.28 (3.79–4.82)
25–29	261,025 (31.5)	514	3.32 (3.05–3.62)
30–34	291,290 (35.1)	522	3.21 (2.94–3.50)
≥ 35	155,406 (18.8)	350	4.27 (3.85–4.74)
Parity			
1	363,542 (43.9)	668	3.28 (3.04–3.54)
2	309,995 (37.4)	618	3.51 (3.25–3.80)
3	110,624 (13.3)	256	3.99 (3.53–4.51)
≥ 4	44,787 (5.4)	146	5.58 (4.75–6.57)
Educational level (years)			
≤ 9	72,647 (8.8)	297	7.44 (6.64–8.34)
10–11	129,519 (15.6)	385	4.35 (3.94–4.81)
12	217,397 (26.2)	398	3.36 (3.04–3.70)
13–14	118,133 (14.3)	208	2.92 (2.55–3.35)
≥ 15	286,624 (34.6)	375	2.50 (2.26–2.76)
Missing	4628 (0.6)	25	13.60 (9.19–20.13)
Country of birth			
Non-Nordic	139,110 (16.8)	460	6.38 (5.82–6.99)
Nordic	689,762 (83.2)	1227	3.08 (2.92–3.26)
Missing	76 (0.0)	1	12.90 (1.82–91.57)
Smoking during pregnancy			
No	713,192 (86.0)	1384	3.48 (3.31–3.67)
Yes	75,094 (9.1)	218	4.55 (3.99–5.20)
Missing	40,662 (4.9)	86	3.45 (2.79–4.26)
Height (cm)			
< 160	105,464 (12.7)	291	4.90 (4.37–5.50)
160–164	211,505 (25.5)	488	4.05 (3.71–4.43)
165–169	242,573 (29.3)	438	3.17 (2.89–3.48)
≥ 170	257,073 (31.0)	437	3.02 (2.75–3.32)
Missing	12,333 (1.5)	34	4.64 (3.31–6.49)
Early pregnancy BMI			
< 18.5	17,261 (2.1)	35	3.57 (2.56–4.97)
18.5–24.9	470,721 (56.8)	830	3.12 (2.92–3.34)
25.0–29.9	179,222 (21.6)	390	3.90 (3.53–4.31)
≥ 30.0	72,825 (8.8)	219	5.63 (4.93–6.43)
Missing	88,919 (10.7)	214	3.84 (3.36–4.39)
Diabetic diseases			
No	820,494 (99.0)	1652	3.55 (3.38–3.72)
Pregestational diabetes	2027 (0.2)	10	9.10 (4.90–16.92)
Gestational diabetes	6427 (0.8)	26	7.66 (5.22–11.25)
Hypertensive diseases			
No	808,555 (97.5)	1632	3.56 (3.39–3.74)
Pregestational hypertension	4197 (0.5)	10	4.80 (2.58–8.92)
Preeclampsia	16,196 (2.0)	46	4.84 (3.62–6.46)
Children			
Sex			
Male	423,549 (51.1)	1083	4.51 (4.25–4.79)
Female	405,399 (48.9)	605	2.63 (2.43–2.85)
Calendar period of delivery			
1998–2001	248,531 (30.0)	845	3.44 (3.22–3.69)
2002–2005	277,384 (33.5)	603	3.67 (3.38–3.97)
2006–2009	303,033 (36.6)	240	3.98 (3.51–4.52)
Onset of labor			
Spontaneous	688,715 (83.1)	1326	3.36 (3.19–3.55)
Induced	133,292 (16.1)	342	4.86 (4.37–5.41)
Missing	6941 (0.8)	20	3.68 (2.38–5.71)
Mode of delivery			
Vaginal non-instrumental	654,010 (78.9)	1267	3.38 (3.20–3.57)
Vaginal instrumental	64,686 (7.8)	143	3.98 (3.37–4.68)
Elective cesarean section	52,469 (6.3)	119	4.35 (3.63–5.20)
Emergency cesarean section	54,583 (6.6)	150	5.12 (4.36–6.00)
Unspecified cesarean section	3200 (0.4)	9	3.81 (1.98–7.33)

^a^Rate is calculated as number of cases per 10,000 person-years

### Statistical analysis

We first calculated crude incidence rates of intellectual disability across categories of maternal and neonatal characteristics.

To assess the association between birth weight percentile and risk of intellectual disability, we calculated standardized incidence rates (SIRs) of intellectual disability across birth weight percentiles (every 10 percentiles), using the distribution of sex and year of delivery of the entire study population as the standard. As our study involved time-to-event data, we performed Cox proportional hazards regression and estimated hazard ratios (HRs) and 95% confidence intervals (CIs) of intellectual disability across the five birth weight percentile groups with 40th–59th as the reference. Ordinary Cox regression was used for population analysis, and stratified Cox regression for sibling comparison analysis. In the sibling comparison analysis, only full siblings discordant for both exposure (i.e., siblings in different percentile groups) and outcome (i.e., siblings with different time-to-event) were informative and thus were included. Attained age was used as the underlying time scale in the Cox models. To assess the potential dose–response pattern across the entire spectrum of birth weight percentiles, we performed an additional analysis by including children born SGA and LGA. To assess the potential non-linear relationship of intellectual disability with birth weight percentile on a continuous scale, we additionally used restricted cubic splines with three knots positioned at the 10th, 50th and 90th percentiles of the distribution of the exposure variable. HRs were estimated using the 50th birth weight percentile as the reference. A similar analytic approach as described above was performed to assess the association between gestational age and risk of intellectual disability.

In population analysis, HRs were estimated after adjustment for maternal age, parity, educational level, country of birth, smoking, height, BMI, diabetic and hypertensive diseases, as well as child’s sex, calendar period of delivery, onset of labor, and mode of delivery. In sibling comparison analysis, adjustment was made for maternal age, parity, smoking, BMI, diabetic and hypertensive diseases, and child’s sex, calendar period of delivery, onset of labor, and mode of delivery. To account for the correlation among full siblings, we used a robust sandwich estimator to correct standard errors in all models.

We estimated the joint impact of birth weight percentile and gestational age with risk of intellectual disability, using children born at 40 weeks and with a birth weight for gestational age from 40th to 59th percentile as the reference category. Only population analysis was performed due to the small numbers of outcomes in the subpopulation of full siblings.

To assess the robustness of the overall associations assessed above, we conducted two sensitivity analyses. First, since we used complete case analysis in the primary analysis, results might have been biased due to missing values of covariates (missing proportions were 0.01%, 0.6%, 0.8%, 1.5%, 4.9%, and 10.7% for maternal country of birth, educational level, onset of labor, height, smoking during pregnancy, and BMI in early pregnancy, respectively). We repeated the Cox regression analysis with missing values imputed through multiple imputation using chained equations. Ten imputations with 50 iterations each were implemented. Second, to improve the validity of diagnosis of intellectual disability, we redefined the outcome as having had an intellectual disability diagnosis in at least two hospital contacts, and repeated both population and sibling comparison analyses.

To evaluate whether the associations of birth weight percentile and gestational age with risk of intellectual disability differed by severity of intellectual disability, we performed secondary analyses where we calculated SIRs and estimated HRs for each type of intellectual disability in both population and sibling comparison analyses.

Data preparation was performed using SAS version 9.4, SAS institute Inc, Cary, NC, USA. Statistical analyses were performed using Stata version 15.1, StataCorp LP, College Station, TX, USA.

## Results

A total of 1688 children were diagnosed with intellectual disability during a median follow-up of 5.5 years (i.e., median age at the end of follow-up: 8.5 years). The median age at diagnosis was 6.1 years (interquartile range: 4.4–8.7 years). The following maternal characteristics were related to higher incidence rates of intellectual disability among children: low (< 25 years) and high (≥ 35 years) age at delivery, increasing parity, lower educational level, non-Nordic origin, smoking during pregnancy, shorter stature, overweight and obesity (BMI 25.0–29.9 and ≥ 30, respectively), and diabetic or hypertensive diseases. Incidence rates were also increased for children delivered in more recent years, for children delivered by induced labor and by cesarean section, and for boys (Table 1). Similar patterns were also shown in the subpopulations of exposure- and outcome-discordant full siblings (Supplementary Tables 2 and 3).

### Primary analysis

**Fig. 1:**
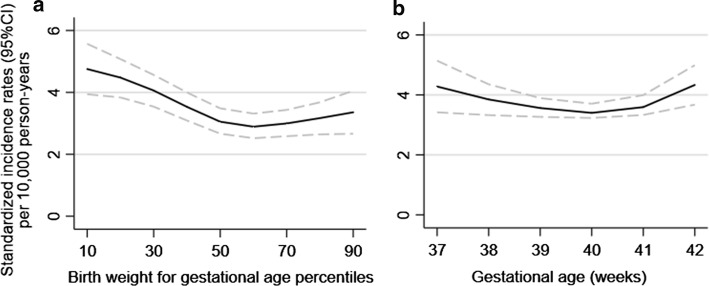
**a** Birth weight for gestational age percentiles and **b** gestational age and standardized incidence rates of any intellectual disability (population analysis). On the X axis of **b**, gestational age of 42 weeks represents 42 weeks and above

For birth weight for gestational age, the SIRs of intellectual disability peaked at the 10th birth weight percentile, then decreased with increasing percentile, but leveled off from 50th percentile and beyond (Fig. 1a). For gestational age, the curve was less pronounced, and the lowest SIR was obtained at 40 weeks (Fig. 1b).

Compared with children born at the 40th–59th birth weight percentiles, children born at the 10th–24th percentiles were, in population analysis, at the highest risk of intellectual disability, but risks were also increased for children at the 25th–39th percentiles (Table 2). Similar estimates were observed in sibling comparison analysis. No differences in risks were observed for children born at larger birth weight percentiles (60th–74th and 75th–90th) versus the reference group. When we also included children born SGA and LGA (< 10th and > 90 percentiles, respectively), a clear dose–response pattern was seen across the entire spectrum of birth weight percentiles for both population analysis and sibling comparison analysis (Supplementary Table 4). In population analysis, children born at 37–39 weeks were at higher risk of intellectual disability than children born at 40 weeks, and children born at 41 weeks or later were also at higher risk (Table 2). Such pattern was not observed in sibling comparison analysis. The smooth curves fitted for the relationships of birth weight percentile (continuous) and gestational age (continuous) with risk of intellectual disability displayed similar patterns (Supplementary Figures 1 and 2 respectively).

**Table 2:** Birth weight for gestational age percentiles and gestational age and risk of intellectual disability in non-malformed, term or post-term, appropriate-for-gestational-age children (complete case analysis) (N = 721,094)

Characteristics	Any intellectual disability					
	Population analysis			Sibling comparison analysis		
	No. of children	No. of cases	HR (95%CI)	No. of children	No. of cases	HR (95%CI)
Birth weight for gestational age percentiles						
Total	721,094	1415		260,928	482	
10th–24th	136,786	338	1.43 (1.22–1.67)	43,641	113	1.52 (1.00–2.31)^a^
25th–39th	141,509	307	1.28 (1.10–1.50)	53,677	120	1.44 (1.00–2.09)^b^
40th–59th	189,271	318	Ref	69,628	103	Ref
60th–74th	132,855	235	1.04 (0.88–1.23)	52,251	86	1.25 (0.79–1.97)
75th–90th	120,673	217	1.03 (0.87–1.23)	41,731	60	1.25 (0.77–2.02)
Gestational age (weeks)						
Total	721,094	1415		245,625	456	
37–38	132,997	282	1.18 (1.00–1.39)^c^	40,987	80	1.33 (0.82–2.16)
39	172,855	340	1.16 (1.00–1.34)^d^	60,831	113	0.96 (0.65–1.41)
40	217,163	367	Ref	72,671	126	Ref
41	140,970	284	1.17 (1.00–1.36)^c^	50,216	92	0.79 (0.51–1.24)
≥ 42	57,109	142	1.23 (1.00–1.50)^e^	20,920	45	1.07 (0.57–2.01)

In population analysis, model was adjusted for maternal age at delivery, parity, educational level, country of birth, smoking during pregnancy, height, BMI in early pregnancy, maternal diabetic and hypertensive diseases, as well as child’s sex, calendar period of delivery, onset of labor, and mode of delivery. In sibling comparison analysis, model was adjusted for maternal age at delivery, parity, smoking during pregnancy, BMI in early pregnancy, maternal diabetic and hypertensive diseases, and child’s sex, calendar period of delivery, onset of labor, and mode of delivery

^a^*P* = 0.050

^b^*P* = 0.053

^c^*P* = 0.054

^d^*P* = 0.041

^e^*P* = 0.049

Table 3 shows the risks of intellectual disability by combinations of birth weight percentiles and gestational age. Compared with the reference group (40th–59th percentiles and 40 weeks’ gestation), children born at 10th–24th and 25th–39th percentiles were at higher risk regardless of length of gestation. Birth weight percentiles between 60th and 74th rendered a pronounced risk increase for children born at 37–38 weeks. Children born at 75th–90th percentiles had a significant risk increase when they were born at 42 weeks or later.

**Table 3:** Birth weight for gestational age percentiles and gestational age and risk of intellectual disability in non-malformed, term or post-term, appropriate-for-gestational-age children, using children born at 40 weeks and with a birth weight for gestational age from 40th to 59th percentile as the reference group (Population analysis, complete case analysis) (N = 721,094)

Gestational age (weeks)	Birth weight for gestational age percentiles									
	10th–24th		25th–39th		40th–59th		60th–74th		75th–90th	
	No. of cases/children	HR (95%CI)	No. of cases/children	HR (95%CI)	No. of cases/children	HR (95%CI)	No. of cases/children	HR (95%CI)	No. of cases/children	HR (95%CI)
37–38	62/23,046	2.01 (1.43–2.84)	49/25,018	1.48 (1.02–2.15)	71/34,514	1.57 (1.12–2.18)	56/25,350	1.66 (1.16–2.37)	44/25,069	1.28 (0.87–1.87)
39	83/31,310	2.08 (1.52–2.86)	70/33,221	1.69 (1.21–2.35)	83/46,100	1.46 (1.06–2.00)	52/32,416	1.28 (0.89–1.83)	52/29,808	1.35 (0.95–1.94)
40	90/40,715	1.74 (1.28–2.38)	84/43,113	1.57 (1.15–2.16)	71/57,161	Ref	62/40,306	1.23 (0.88–1.73)	60/35,868	1.30 (0.93–1.84)
41	68/28,671	1.82 (1.31–2.54)	75/28,090	2.10 (1.52–2.91)	57/36,890	1.21 (0.86–1.72)	47/25,236	1.45 (1.00–2.10)^a^	37/22,083	1.28 (0.86–1.90)
≥ 42	35/13,044	1.72 (1.15–2.59)	29/12,067	1.60 (1.04–2.47)	36/14,606	1.66 (1.11–2.50)	18/9547	1.27 (0.75–2.13)	24/7845	2.02 (1.26–3.23)

Model was adjusted for interaction between gestational age and birth weight for gestational age percentile, maternal age at delivery, parity, educational level, country of birth, smoking during pregnancy, height, BMI in early pregnancy, maternal diabetic and hypertensive diseases, as well as child’s sex, calendar period of delivery, onset of labor, and mode of delivery

^a^*P* = 0.048

### Sensitivity analysis

Relative to the complete case analysis, similar overall associations of birth weight percentile and gestational age with risk of intellectual disability were observed after multiple imputation (Supplementary Table 5). Analyses where outcome was redefined as at least two diagnoses of intellectual disability also provided similar results regarding birth weight percentiles but showed weaker associations regarding gestational age (Supplementary Table 6).

### Secondary analysis

Among all children with intellectual disability, 47% (n = 787) had mild impairment. The SIRs of intellectual disability by severity across birth weight percentiles and gestational age are shown in Supplementary Figures 3 and 4 respectively. Similar to the overall association for any intellectual disability, the risk of mild intellectual disability was higher among children born at the 10th–24th and 25th–39th percentiles in population analysis and even higher in sibling comparison analysis. Risks of moderate and severe, other or unspecified types of intellectual disability were only significantly increased in children born at the 10th–24th percentiles in population analysis (Supplementary Table 7). Risks of mild and other or unspecified types of intellectual disability were higher in children born at 37–38 weeks, whereas risks of moderate and severe intellectual disability were higher in children born at 42 weeks or later in population analysis (Supplementary Table 8).

## Discussion

In this nationwide population-based study of non-malformed, term or post-term, AGA children, we found that children born with lower birth weight percentiles had a higher risk of intellectual disability, both when compared with the general population, and with their siblings. A weak U-shaped association between gestational age and intellectual disability was observed in population analysis, indicating that children born early term or post-term had a higher risk of intellectual disability, although such pattern was not observed among siblings. The increased risk for low birth weight percentiles was stable irrespective of gestational age. Children with higher birth weight percentiles were at higher risk of intellectual disability if they were born post-term.

In agreement with previous studies [35], we found that children born SGA were at increased risk of intellectual disability. However, few studies, including no sibling design studies, have investigated the association between fetal growth and intellectual disability among AGA children. A previous Swedish study reported that birth weight for gestational age z-score was positively associated with intellectual performance among young men born at term, but did not examine differences between AGA and SGA or LGA [36]. A similar study on young Norwegian men showed that low birth weight for gestational age z-score was associated with higher risk of poor intellectual performance, but high z-score (except > 3.0) was not associated with intellectual performance [37]. One study focusing on children noted that fetal growth, as assessed by percentage of optimal birth weight, was not associated with intellectual disability in AGA children [5]. This null finding may be due to differences in study population characteristics, statistical methods, and sample size. Our null results regarding higher birth weight percentiles seem to align with one previous study on intellectual capacity among LGA children compared with AGA children [38], but further studies are required to validate that higher birth weight percentiles in AGA children are not associated with risk of intellectual disability.

A number of studies have examined the association between gestational age and cognitive level in children born at term or post-term [12, 36, 39]. Our findings align with most of previous studies, which showed that early term or post-term birth was associated with a higher risk of intellectual disability, special education needs, and lower IQ scores [12, 36, 39–41]. The increased risk for children born post-term might indicate that a failure to be born full term (39–40 weeks) might relate to other congenital developmental conditions or that perinatal asphyxia related to post-term birth might have an adverse impact on brain development compared to full term birth [42]. A recent Swedish study, including the full range of birth weight percentiles, found an evident U-shaped association between gestational age and intellectual disability among siblings [12]. Our findings of non-significant associations in sibling design, which were based on a larger population (national data) and included only AGA children, might not be directly comparable but provide complementary evidence on variation in risk of intellectual disability among children born with appropriate weight and term gestation.

In this study, sibling comparison design was applied to control for unmeasured confounding factors shared by siblings. While associations regarding birth weight percentile persisted in sibling comparison analysis, associations for gestational age attenuated. This supports that variation in gestational age might be explained more by maternal genetic effect and shared (sibling) environment than other phenotypes such as birth weight [43]. Nevertheless, null findings may also imply an effect mediated only through familiar environment, which is completely “controlled away” in sibling comparison analysis [44].

The risk increase for lower birth weight percentile did not differ within the range of gestation age (≥ 37 weeks). This suggests that even for full term infants, failure to reach their optimal growth potential may increase the tendency for intellectual and adaptive difficulties, potentially resulted from, e.g., reduced total brain volume [45, 46]. On the contrary, higher birth weight percentiles were associated with higher risk of intellectual disability for post-term children, although not significant for 60th–74th percentiles, lending further support for interventions to deliver infants if pregnancy prolongation is considered hazardous [42].

Mild intellectual disability accounted for almost half of the cases and shows similar associations as any intellectual disability. The higher risk of mild intellectual disability for lower birth weight percentiles in sibling comparison analysis versus population analysis could, however, be a chance finding. The largely non-significant results for more severe and other types of intellectual disability may be attributed to lack of statistical power.

A strength of the present study is the use of prospectively and independently collected information on exposures, outcome, and covariates based on Swedish national registries, which yielded sufficient statistical power to evaluate the risk of intellectual disability. Ultrasonography was performed to the majority of women (87% of births) which ensured optimal pregnancy dating. In addition to population analysis, we applied sibling comparison analysis to adjust associations for unmeasured genetic and environment factors shared by siblings. Although restriction to siblings might induce selection bias, this might be less of a concern in the present study which showed similar distributions of characteristics between the entire study population and the subpopulations of exposure- and outcome-discordant siblings. Some potential limitations deserve mentioning. Complete case analysis may have induced bias due to missing covariate information, but the multiple imputation analysis, despite the assumption of missing data at random [47], provided similar and reassuring results. Residual confounding due to unmeasured confounders, such as maternal nutrition and parental intelligence, is possible [7, 48]. Multiple testing can lead to false positives but might be less of a problem in this study, as our main analysis only involved five statistical tests (Tables 2 and 3). We did not have sufficient statistical power to explore the joint impact of birth weight percentile and gestational age in the sibling design.

In conclusion, lower birth weight percentiles are associated with higher risks of intellectual disability in AGA children born term or post-term, irrespective of gestational age. This study enriches the knowledge of short- and long-term neurological outcomes of non-optimal fetal growth in AGA infants [9].

## Electronic supplementary material

Below is the link to the electronic supplementary material.

Supplementary material 1 (DOCX 4279 kb)
