# Supplementary material for: Risk of intellectual disability in children born appropriate-for-gestational-age at term or post-term: impact of birth weight for gestational age and gestational age
Source: Eur J Epidemiol. 2019 Dec 2;35(3):273–82. doi: 10.1007/s10654-019-00590-7 (PMC7154017; doi:10.1007/s10654-019-00590-7)
Supplement: Supplementary file 1 — Supplementary material 1 (DOCX 4279 kb) [file 10654_2019_590_MOESM1_ESM.docx]

**Supplementary material**

**Risk of intellectual disability in children born appropriate-for-gestational-age at term or post-term: impact of birth weight for gestational age and gestational age**

Ruoqing Chen^1^ ∙ Kristina Tedroff ^2^ ∙ Eduardo Villamor ^3^ ∙ Donghao Lu^4,5,6^ ∙ Sven Cnattingius^1^

**Affiliations**

^1^ Clinical Epidemiology Division, Department of Medicine Solna, Karolinska Institutet, Stockholm, Sweden

^2^ Department of Women's and Children's Health, Karolinska Institutet, Stockholm, Sweden

^3^ Department of Epidemiology, School of Public Health, University of Michigan, Ann Arbor, MI, USA

^4^ Department of Medical Epidemiology and Biostatistics, Karolinska Institutet, Stockholm, Sweden

^5^ Channing Division of Network Medicine, Brigham and Women’s Hospital and Harvard Medical School, Boston, MA, USA

^6^ Department of Epidemiology, Harvard T.H. Chan School of Public Health, Boston, MA, USA

**Corresponding author:** Ruoqing Chen, Clinical Epidemiology Division, Department of Medicine Solna, Karolinska Institutet, 171 76 Stockholm, Sweden. Email: [ruoqing.chen@ki.se](mailto:ruoqing.chen@ki.se).

Supplementary Figure 1. Birth weight for gestational age percentiles and risk of intellectual disability in non-malformed, term or post-term, appropriate-for-gestational-age children (A restricted cubic spline transformation with 3 knots was used to model the non-linearity in this association). In population analysis, model was adjusted for maternal age at delivery, parity, educational level, country of birth, smoking during pregnancy, height, BMI in early pregnancy, maternal diabetic and hypertensive diseases, as well as child’s sex, calendar period of delivery, onset of labor, and mode of delivery. In sibling comparison analysis, model was adjusted for maternal age at delivery, parity, smoking during pregnancy, BMI in early pregnancy, maternal diabetic and hypertensive diseases, and child’s sex, calendar period of delivery, onset of labor, and mode of delivery.


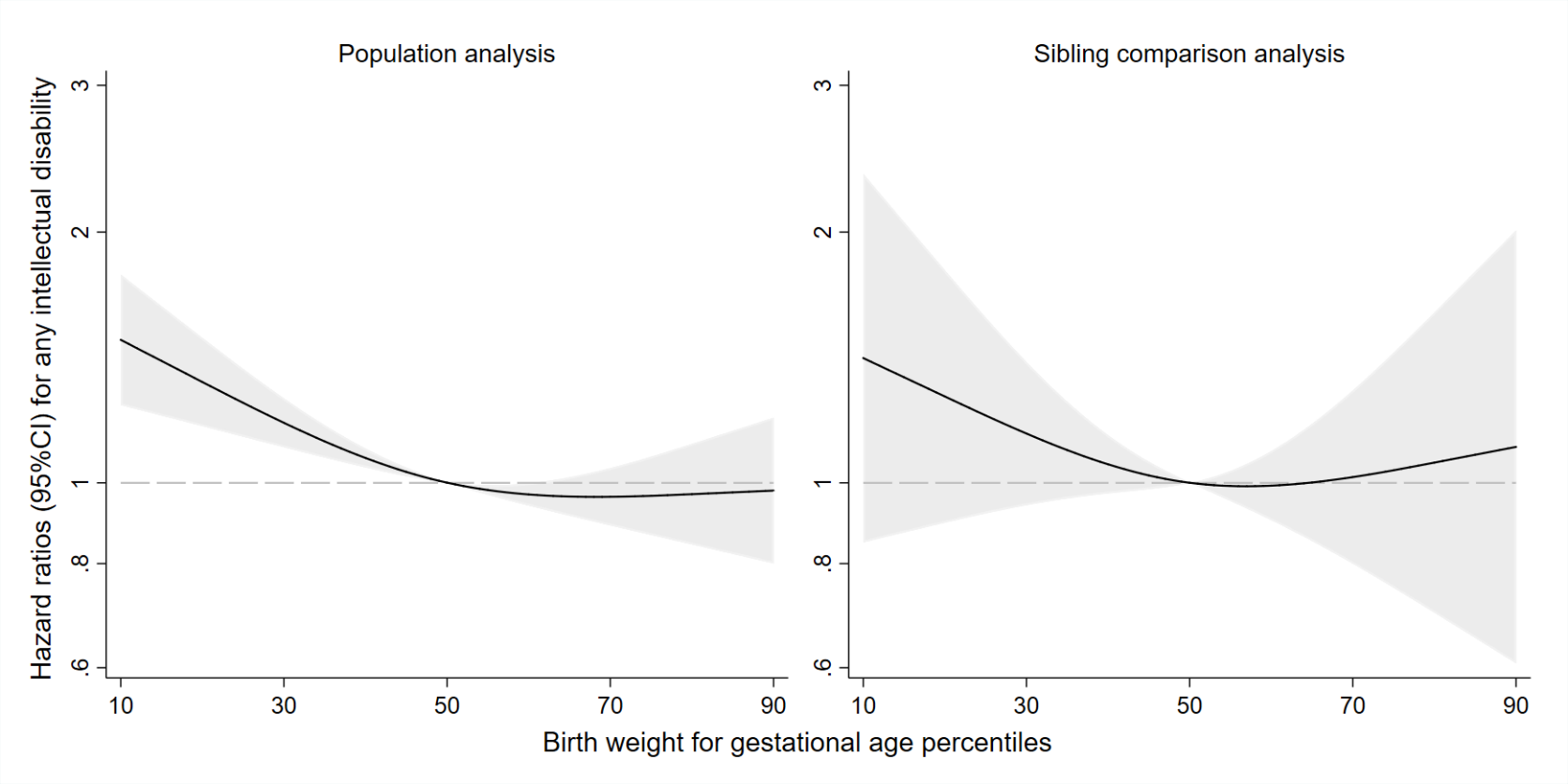


Supplementary Figure 2. Gestational age and risk of intellectual disability in non-malformed, term or post-term, appropriate-for-gestational-age children (A restricted cubic spline transformation with 3 knots was used to model the non-linearity in this association). In population analysis, model was adjusted for maternal age at delivery, parity, educational level, country of birth, smoking during pregnancy, height, BMI in early pregnancy, maternal diabetic and hypertensive diseases, as well as child’s sex, calendar period of delivery, onset of labor, and mode of delivery. In sibling comparison analysis, model was adjusted for maternal age at delivery, parity, smoking during pregnancy, BMI in early pregnancy, maternal diabetic and hypertensive diseases, and child’s sex, calendar period of delivery, onset of labor, and mode of delivery. On the X axis of the graphs, gestational age of 42 weeks represents 42 weeks and above.


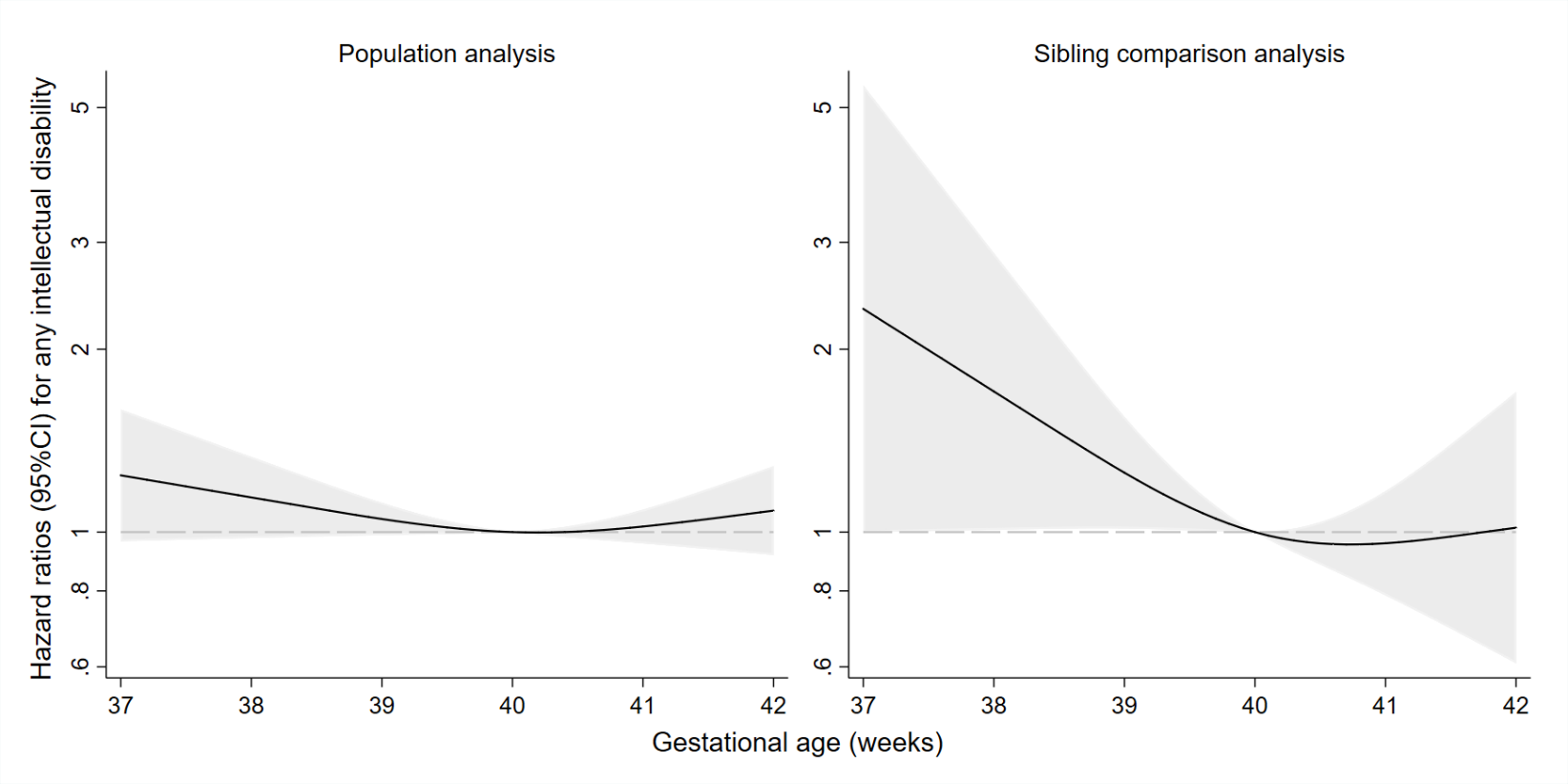


Supplementary Figure 3. Birth weight for gestational age percentiles and standardized incidence rates of intellectual disability (ID) of different severity (population analysis). The incidence rates were standardized according to the distribution of sex and calendar period of delivery of the entire study population.


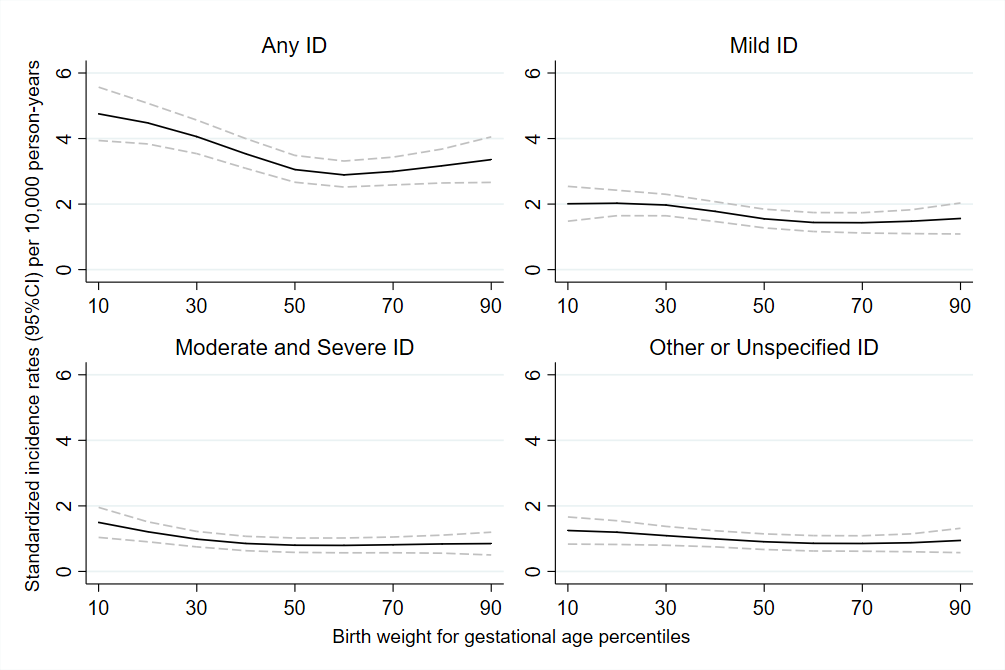


Supplementary Figure 4. Gestational age and standardized incidence rates of intellectual disability (ID) of different severity (population analysis). The incidence rates were standardized according to the distribution of sex and calendar period of delivery of the entire study population. On the X axis of the graphs, gestational age of 42 weeks represents 42 weeks and above.


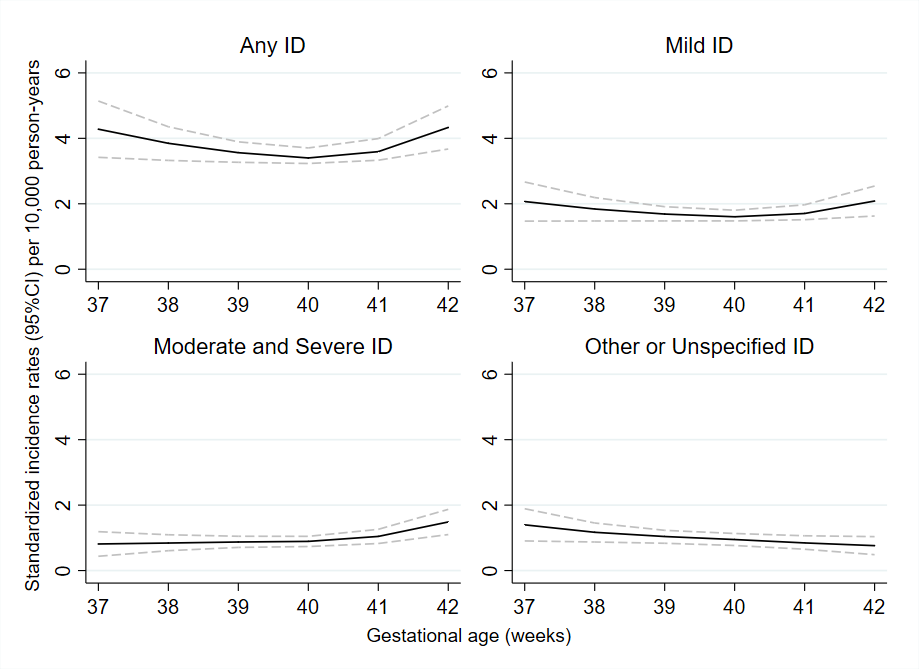


| **Supplementary Table 1. ICD codes of maternal and child diseases** | |
| --- | --- |
| **Diagnoses** | **ICD-10 codes** |
| Major malformations | Q00-Q99, excluding^a^ Q17.0, Q17.5, Q18.0, Q18.1, Q25.0, Q27.0, Q31.5, Q32.0, Q38.1, Q52.3, Q53.0-.9, Q65.0-.9, Q66.5-.9, Q69.0, Q69.9, Q70.3, Q76.0, Q79.9, Q82.5, Q82.9 |
| Intellectual disability |  |
| Mild | F70 |
| Moderate and severe | F71-F73 |
| Other or unspecified | F78-F79 |
| Maternal diabetic diseases | E10-E14, O24.0-O24.4 |
| Maternal hypertensive diseases | O10-O11, O14-O15, I10-I15 |
| ^a^ The following ICD-10 codes represent diagnoses of minor malformations, defined by the Swedish National Board of Health and Welfare (see https://www.socialstyrelsen.se/globalassets/sharepoint-dokument/dokument-webb/ovrigt/diagnoser-som-inte-ska-rapporteras-om-fosterskador.pdf). | |

| **Supplementary Table 2. Maternal and neonatal characteristics and rate of any intellectual disability in term or post-term children with appropriate birth weight for gestational age (Birth weight percentile and outcome discordant siblings, N=329,550)** | | | |
| --- | --- | --- | --- |
| **Characteristics** | **No. of children (N[%])** | **Intellectual disability** | |
|  |  | **No. of cases** | **Rate (95%CI)^a^** |
| Total | 329550 | 616 | 3.28 (3.03-3.55) |
| Mothers |  |  |  |
| Age at child's birth (years) |  |  |  |
| <20 | 3582 (1.1) | 12 | 4.63 (2.63-8.15) |
| 20-24 | 41232 (12.5) | 120 | 4.34 (3.63-5.19) |
| 25-29 | 110935 (33.7) | 192 | 2.76 (2.40-3.18) |
| 30-34 | 120653 (36.6) | 202 | 3.13 (2.73-3.60) |
| ≥35 | 53148 (16.1) | 90 | 3.81 (3.10-4.68) |
| Parity |  |  |  |
| 1 | 123221 (37.4) | 239 | 2.71 (2.39-3.08) |
| 2 | 139320 (42.3) | 236 | 3.41 (3.01-3.88) |
| 3 | 46994 (14.3) | 86 | 4.10 (3.32-5.06) |
| ≥4 | 20015 (6.1) | 55 | 5.76 (4.42-7.50) |
| Educational level (years) |  |  |  |
| ≤9 | 25899 (7.9) | 115 | 7.96 (6.63-9.56) |
| 10-11 | 44677 (13.6) | 105 | 3.61 (2.98-4.37) |
| 12 | 87074 (26.4) | 148 | 3.05 (2.59-3.58) |
| 13-14 | 46568 (14.1) | 76 | 2.72 (2.18-3.41) |
| ≥15 | 123773 (37.6) | 160 | 2.38 (2.04-2.78) |
| Missing | 1559 (0.5) | 12 | 17.21 (9.77-30.30) |
| Country of birth |  |  |  |
| Non-Nordic | 52418 (15.9) | 199 | 6.95 (6.05-7.99) |
| Nordic | 277108 (84.1) | 417 | 2.62 (2.38-2.88) |
| Missing | 24 (0.0) | 0 | N/A |
| Smoking during pregnancy |  |  |  |
| No | 290803 (88.2) | 537 | 3.27 (3.01-3.56) |
| Yes | 22191 (6.7) | 53 | 3.80 (2.90-4.97) |
| Missing | 16556 (5.0) | 26 | 2.67 (1.82-3.92) |
| Height (cm) |  |  |  |
| <160 | 40510 (12.3) | 112 | 4.86 (4.03-5.84) |
| 160-164 | 85020 (25.8) | 189 | 3.89 (3.37-4.48) |
| 165-169 | 98667 (29.9) | 166 | 2.95 (2.53-3.43) |
| ≥170 | 103105 (31.3) | 145 | 2.48 (2.11-2.92) |
| Missing | 2248 (0.7) | 4 | 2.94 (1.11-7.85) |
| Early pregnancy BMI |  |  |  |
| <18.5 | 6521 (2.0) | 13 | 3.29 (1.91-5.67) |
| 18.5-24.9 | 190010 (57.7) | 309 | 2.83 (2.53-3.16) |
| 25.0-29.9 | 70668 (21.4) | 160 | 4.11 (3.52-4.80) |
| ≥30.0 | 27633 (8.4) | 64 | 4.48 (3.51-5.72) |
| Missing | 34718 (10.5) | 70 | 3.28 (2.59-4.14) |
| Diabetic diseases |  |  |  |
| No | 326956 (99.2) | 606 | 3.25 (3.00-3.52) |
| Pregestational diabetes | 474 (0.1) | 1 | 4.20 (0.59-29.83) |
| Gestational diabetes | 2120 (0.6) | 9 | 8.30 (4.32-15.95) |
| Hypertensive diseases |  |  |  |
| No | 322823 (98.0) | 608 | 3.31 (3.06-3.58) |
| Pregestational hypertension | 1399 (0.4) | 2 | 2.97 (0.74-11.88) |
| Preeclampsia | 5328 (1.6) | 6 | 1.73 (0.78-3.85) |
|  |  |  |  |
| Children |  |  |  |
| Sex |  |  |  |
| Male | 168925 (51.3) | 387 | 4.02 (3.64-4.44) |
| Female | 160625 (48.7) | 229 | 2.50 (2.20-2.85) |
| Calendar period of delivery |  |  |  |
| 1998-2001 | 87176 (26.5) | 271 | 3.20 (2.84-3.61) |
| 2002-2005 | 133568 (40.5) | 269 | 3.39 (3.01-3.82) |
| 2006-2009 | 108806 (33.0) | 76 | 3.17 (2.53-3.97) |
| Onset of labor |  |  |  |
| Spontaneous | 278955 (84.6) | 515 | 3.21 (2.94-3.50) |
| Induced | 48102 (14.6) | 97 | 3.81 (3.13-4.65) |
| Missing | 2493 (0.8) | 4 | 2.11 (0.79-5.62) |
| Mode of delivery |  |  |  |
| Vaginal noninstrumental | 269300 (81.7) | 485 | 3.18 (2.91-3.48) |
| Vaginal instrumental | 22409 (6.8) | 52 | 3.56 (2.71-4.67) |
| Elective cesarean section | 19161 (5.8) | 36 | 3.79 (2.74-5.26) |
| Emergency cesarean section | 17575 (5.3) | 43 | 4.05 (3.00-5.46) |
| Unspecified cesarean section | 1105 (0.3) | 0 | N/A |
| ^a^ Rate is calculated as number of cases per 10000 person-years. | | | |

| **Supplementary Table 3. Maternal and neonatal characteristics and rate of any intellectual disability in term or post-term children with appropriate birth weight for gestational age (Gestational age and outcome discordant siblings, N=309,983)** | | | |
| --- | --- | --- | --- |
| **Characteristics** | **No. of children (N[%])** | **Intellectual disability** | |
|  |  | **No. of cases** | **Rate (95%CI)^a^** |
| Total | 309983 | 584 | 3.30 (3.04-3.58) |
| Mothers |  |  |  |
| Age at child's birth (years) |  |  |  |
| <20 | 3319 (1.1) | 13 | 5.39 (3.13-9.28) |
| 20-24 | 38918 (12.6) | 104 | 3.97 (3.27-4.81) |
| 25-29 | 104215 (33.6) | 204 | 3.12 (2.72-3.58) |
| 30-34 | 113684 (36.7) | 183 | 3.01 (2.61-3.48) |
| ≥35 | 49847 (16.1) | 80 | 3.62 (2.91-4.51) |
| Parity |  |  |  |
| 1 | 115988 (37.4) | 237 | 2.85 (2.51-3.24) |
| 2 | 130715 (42.2) | 216 | 3.33 (2.91-3.80) |
| 3 | 44329 (14.3) | 79 | 3.99 (3.20-4.98) |
| ≥4 | 18951 (6.1) | 52 | 5.75 (4.38-7.55) |
| Educational level (years) |  |  |  |
| ≤9 | 24446 (7.9) | 104 | 7.64 (6.30-9.26) |
| 10-11 | 42382 (13.7) | 113 | 4.09 (3.40-4.92) |
| 12 | 81522 (26.3) | 140 | 3.07 (2.60-3.62) |
| 13-14 | 43866 (14.2) | 77 | 2.93 (2.34-3.66) |
| ≥15 | 116229 (37.5) | 140 | 2.22 (1.88-2.62) |
| Missing | 1538 (0.5) | 10 | 14.32 (7.70-26.61) |
| Country of birth |  |  |  |
| Non-Nordic | 49467 (16.0) | 181 | 6.69 (5.79-7.74) |
| Nordic | 260497 (84.0) | 403 | 2.69 (2.44-2.97) |
| Missing | 19 (0.0) | 0 | N/A |
| Smoking during pregnancy |  |  |  |
| No | 273418 (88.2) | 513 | 3.32 (3.04-3.62) |
| Yes | 21138 (6.8) | 47 | 3.54 (2.66-4.71) |
| Missing | 15427 (5.0) | 24 | 2.64 (1.77-3.94) |
| Height (cm) |  |  |  |
| <160 | 38400 (12.4) | 112 | 5.11 (4.24-6.15) |
| 160-164 | 80017 (25.8) | 168 | 3.68 (3.16-4.28) |
| 165-169 | 92785 (29.9) | 146 | 2.75 (2.34-3.24) |
| ≥170 | 96715 (31.2) | 155 | 2.81 (2.40-3.29) |
| Missing | 2066 (0.7) | 3 | 2.37 (0.77-7.36) |
| Early pregnancy BMI |  |  |  |
| <18.5 | 6131 (2.0) | 12 | 3.22 (1.83-5.68) |
| 18.5-24.9 | 178825 (57.7) | 290 | 2.81 (2.51-3.15) |
| 25.0-29.9 | 66362 (21.4) | 147 | 4.03 (3.42-4.73) |
| ≥30.0 | 26173 (8.4) | 68 | 5.02 (3.96-6.36) |
| Missing | 32492 (10.5) | 67 | 3.36 (2.64-4.27) |
| Diabetic diseases |  |  |  |
| No | 307649 (99.2) | 574 | 3.27 (3.01-3.55) |
| Pregestational diabetes | 392 (0.1) | 2 | 9.61 (2.40-38.41) |
| Gestational diabetes | 1942 (0.6) | 8 | 8.00 (4.00-15.99) |
| Hypertensive diseases |  |  |  |
| No | 303710 (98.0) | 574 | 3.32 (3.06-3.60) |
| Pregestational hypertension | 1288 (0.4) | 1 | 1.59 (0.22-11.28) |
| Preeclampsia | 4985 (1.6) | 9 | 2.78 (1.44-5.34) |
|  |  |  |  |
| Children |  |  |  |
| Sex |  |  |  |
| Male | 159325 (51.4) | 368 | 4.04 (3.65-4.48) |
| Female | 150658 (48.6) | 216 | 2.51 (2.20-2.87) |
| Calendar period of delivery |  |  |  |
| 1998-2001 | 82045 (26.5) | 259 | 3.25 (2.88-3.67) |
| 2002-2005 | 125932 (40.6) | 267 | 3.57 (3.17-4.02) |
| 2006-2009 | 102006 (32.9) | 58 | 2.58 (1.99-3.34) |
| Onset of labor |  |  |  |
| Spontaneous | 261613 (84.4) | 486 | 3.22 (2.94-3.52) |
| Induced | 46060 (14.9) | 94 | 3.89 (3.18-4.76) |
| Missing | 2310 (0.7) | 4 | 2.29 (0.86-6.10) |
| Mode of delivery |  |  |  |
| Vaginal noninstrumental | 252062 (81.3) | 459 | 3.21 (2.93-3.52) |
| Vaginal instrumental | 21486 (6.9) | 48 | 3.42 (2.58-4.54) |
| Elective cesarean section | 17819 (5.7) | 36 | 4.18 (3.02-5.80) |
| Emergency cesarean section | 17635 (5.7) | 41 | 3.82 (2.81-5.19) |
| Unspecified cesarean section | 981 (0.3) | 0 | N/A |
| ^a^ Rate is calculated as number of cases per 10000 person-years. | | | |

| **Supplementary Table 4. Birth weight for gestational age percentiles and risk of intellectual disability in non-malformed, term or post-term children (complete case analysis) (N=877407)** | | | | | | | |
| --- | --- | --- | --- | --- | --- | --- | --- |
| **Characteristic** | **Any intellectual disability** | | | | | | |
|  | **Population analysis** | | |  | **Sibling comparison analysis** | | |
|  | **No. of children** | **No. of cases** | **HR (95%CI)** |  | **No. of children** | **No. of cases** | **HR (95%CI)** |
| **Birth weight for gestational age percentiles** |  |  |  |  |  |  |  |
| Total | 877407 | 1914 |  |  | 372170 | 783 |  |
| <10^th^ | 70511 | 317 | 2.44 (2.07-2.86) |  | 25985 | 128 | 2.35 (1.48-3.72) |
| 10^th^ -24^th^ | 136786 | 338 | 1.45 (1.24-1.69) |  | 54540 | 140 | 1.72 (1.18-2.53) |
| 25^th^ -39^th^ | 141509 | 307 | 1.29 (1.10-1.51) |  | 61945 | 139 | 1.52 (1.07-2.15) |
| 40^th^ -59^th^ | 189271 | 318 | Ref |  | 80262 | 131 | Ref |
| 60^th^ -74^th^ | 132855 | 235 | 1.04 (0.87-1.23) |  | 61607 | 104 | 1.03 (0.68-1.56) |
| 75^th^ -90^th^ | 120673 | 217 | 1.02 (0.86-1.21) |  | 54598 | 83 | 0.89 (0.58-1.37) |
| >90^th^ | 85802 | 182 | 1.08 (0.89-1.30) |  | 33233 | 58 | 0.86 (0.54-1.36) |
| In population analysis, model was adjusted for maternal age at delivery, parity, educational level, country of birth, smoking during pregnancy, height, BMI in early pregnancy, maternal diabetic and hypertensive diseases, as well as child’s sex, calendar period of delivery, onset of labor, and mode of delivery. | | | | | | | |
| In sibling comparison analysis, model was adjusted for maternal age at delivery, parity, smoking during pregnancy, BMI in early pregnancy, maternal diabetic and hypertensive diseases, and child’s sex, calendar period of delivery, onset of labor, and mode of delivery. | | | | | | | |

| **Supplementary Table 5. Birth weight for gestational age percentiles and gestational age and risk of intellectual disability in non-malformed, term or post-term, appropriate-for-gestational-age children (multiple imputation analysis) (N=828948)** | | | | | | | |
| --- | --- | --- | --- | --- | --- | --- | --- |
| **Characteristics** | **Any intellectual disability** | | | | | | |
|  | **Population analysis** | | |  | **Sibling comparison analysis** | | |
|  | **No. of children** | **No. of cases** | **HR (95%CI)** |  | **No. of children** | **No. of cases** | **HR (95%CI)** |
| **Birth weight for gestational age percentiles** |  |  |  |  |  |  |  |
| Total | 828948 | 1688 |  |  | 329550 | 616 |  |
| 10^th^ -24^th^ | 156932 | 407 | 1.43 (1.24-1.65) |  | 58322 | 118 | 1.51 (1.03-2.20) |
| 25^th^ -39^th^ | 162578 | 364 | 1.26 (1.09-1.46) |  | 80422 | 142 | 1.43 (1.02-2.00) |
| 40^th^ -59^th^ | 217463 | 383 | Ref |  | 101159 | 174 | Ref |
| 60^th^ -74^th^ | 152817 | 275 | 1.01 (0.87-1.18) |  | 64681 | 126 | 1.10 (0.75-1.62) |
| 75^th^ -90^th^ | 139158 | 259 | 1.02 (0.87-1.19) |  | 24966 | 56 | 1.17 (0.78-1.77) |
| **Gestational age (weeks)** |  |  |  |  |  |  |  |
| Total | 828948 | 1688 |  |  | 309983 | 584 |  |
| 37-38 | 155560 | 341 | 1.13 (0.97-1.32) |  | 52505 | 102 | 1.18 (0.80-1.77) |
| 39 | 198625 | 408 | 1.15 (1.01-1.32) |  | 76780 | 145 | 0.96 (0.69-1.35) |
| 40 | 248566 | 438 | Ref |  | 91223 | 155 | Ref |
| 41 | 161003 | 332 | 1.15 (0.99-1.33) |  | 63102 | 122 | 0.99 (0.69-1.44) |
| ≥42 | 65194 | 169 | 1.21 (1.01-1.46) |  | 26373 | 60 | 1.19 (0.70-2.02) |
| In population analysis, model was adjusted for maternal age at delivery, parity, educational level, country of birth, smoking during pregnancy, height, BMI in early pregnancy, maternal diabetic and hypertensive diseases, as well as child’s sex, calendar period of delivery, onset of labor, and mode of delivery. | | | | | | | |
| In sibling comparison analysis, model was adjusted for maternal age at delivery, parity, smoking during pregnancy, BMI in early pregnancy, maternal diabetic and hypertensive diseases, and child’s sex, calendar period of delivery, onset of labor, and mode of delivery. | | | | | | | |

| **Supplementary Table 6. Birth weight for gestational age percentiles and gestational age and risk of intellectual disability (at least two diagnoses) in non-malformed, term or post-term, appropriate-for-gestational-age children (complete case analysis) (N=721094)** | | | | | | | |
| --- | --- | --- | --- | --- | --- | --- | --- |
| **Characteristics** | **Any intellectual disability** | | | | | | |
|  | **Population analysis** | | |  | **Sibling comparison analysis** | | |
|  | **No. of children** | **No. of cases** | **HR (95%CI)** |  | **No. of children** | **No. of cases** | **HR (95%CI)** |
| **Birth weight for gestational age percentiles** |  |  |  |  |  |  |  |
| Total | 721094 | 987 |  |  | 260928 | 352 |  |
| 10^th^ -24^th^ | 136786 | 237 | 1.45 (1.21-1.75) |  | 43641 | 82 | 1.56 (0.96-2.55) |
| 25^th^ -39^th^ | 141509 | 214 | 1.29 (1.07-1.56) |  | 53677 | 84 | 1.56 (1.01-2.42) |
| 40^th^ -59^th^ | 189271 | 221 | Ref |  | 69628 | 77 | Ref |
| 60^th^ -74^th^ | 132855 | 173 | 1.10 (0.90-1.34) |  | 52251 | 69 | 1.50 (0.90-2.49) |
| 75^th^ -90^th^ | 120673 | 142 | 0.97 (0.78-1.20) |  | 41731 | 40 | 1.21 (0.69-2.11) |
| **Gestational age (weeks)** |  |  |  |  |  |  |  |
| Total | 721094 | 987 |  |  | 245625 | 328 |  |
| 37-38 | 132997 | 194 | 1.12 (0.91-1.36) |  | 40987 | 59 | 1.39 (0.78-2.47) |
| 39 | 172855 | 226 | 1.08 (0.90-1.29) |  | 60831 | 78 | 1.01 (0.65-1.57) |
| 40 | 217163 | 261 | Ref |  | 72671 | 91 | Ref |
| 41 | 140970 | 206 | 1.19 (0.99-1.43) |  | 50216 | 67 | 0.74 (0.44-1.23) |
| ≥42 | 57109 | 100 | 1.22 (0.95-1.55) |  | 20920 | 33 | 1.07 (0.52-2.19) |
| In population analysis, model was adjusted for maternal age at delivery, parity, educational level, country of birth, smoking during pregnancy, height, BMI in early pregnancy, maternal diabetic and hypertensive diseases, as well as child’s sex, calendar period of delivery, onset of labor, and mode of delivery. | | | | | | | |
| In sibling comparison analysis, model was adjusted for maternal age at delivery, parity, smoking during pregnancy, BMI in early pregnancy, maternal diabetic and hypertensive diseases, and child’s sex, calendar period of delivery, onset of labor, and mode of delivery. | | | | | | | |

| **Supplementary Table 7. Birth weight for gestational age percentiles and risk of intellectual disability of different severity in non-malformed, term or post-term, appropriate-for-gestational-age children (Complete case analysis) (N=721094)** | | | | | | | | | |
| --- | --- | --- | --- | --- | --- | --- | --- | --- | --- |
| **Birth weight for gestational age percentiles** | **Population analysis** | | | | | | | | |
|  | **No. of children** | **Mild** | |  | **Moderate and Severe** | |  | **Other or unspecified** | |
|  |  | **No. of cases** | **HR (95%CI)** |  | **No. of cases** | **HR (95%CI)** |  | **No. of cases** | **HR (95%CI)** |
| Total | 721094 | 651 |  |  | 369 |  |  | 395 |  |
| 10^th^ -24^th^ | 136786 | 144 | 1.27 (1.01-1.61) |  | 98 | 1.82 (1.35-2.47) |  | 96 | 1.37 (1.02-1.82) |
| 25^th^ -39^th^ | 141509 | 150 | 1.33 (1.06-1.67) |  | 73 | 1.32 (0.95-1.82) |  | 84 | 1.18 (0.88-1.59) |
| 40^th^ -59^th^ | 189271 | 149 | Ref |  | 75 | Ref |  | 94 | Ref |
| 60^th^ -74^th^ | 132855 | 106 | 1.01 (0.79-1.30) |  | 70 | 1.30 (0.94-1.80) |  | 59 | 0.88 (0.64-1.22) |
| 75^th^ -90^th^ | 120673 | 102 | 1.05 (0.82-1.35) |  | 53 | 1.04 (0.73-1.48) |  | 62 | 1.00 (0.72-1.38) |
|  | **Sibling comparison analysis** | | | | | | | | |
|  | **No. of children** | **Mild** | |  | **Moderate and Severe** | |  | **Other or unspecified** | |
|  |  | **No. of cases** | **HR (95%CI)** |  | **No. of cases** | **HR (95%CI)** |  | **No. of cases** | **HR (95%CI)** |
| Total | 260928 | 216 |  |  | 134 |  |  | 132 |  |
| 10^th^ -24^th^ | 43641 | 46 | 2.41 (1.05-5.51) |  | 37 | 2.76 (0.91-8.36) |  | 30 | 1.42 (0.62-3.26) |
| 25^th^ -39^th^ | 53677 | 62 | 2.37 (1.25-4.49) |  | 27 | 1.35 (0.59-3.09) |  | 31 | 0.87 (0.38-2.00) |
| 40^th^ -59^th^ | 69628 | 44 | Ref |  | 31 | Ref |  | 28 | Ref |
| 60^th^ -74^th^ | 52251 | 35 | 0.89 (0.43-1.86) |  | 29 | 2.71 (0.85-8.69) |  | 22 | 1.06 (0.42-2.64) |
| 75^th^ -90^th^ | 41731 | 29 | 2.01 (0.77-5.23) |  | 10 | 0.50 (0.19-1.29) |  | 21 | 1.59 (0.52-4.90) |
| In population analysis, model was adjusted for maternal age at delivery, parity, educational level, country of birth, smoking during pregnancy, height, BMI in early pregnancy, maternal diabetic and hypertensive diseases, as well as child’s sex, calendar period of delivery, onset of labor, and mode of delivery. | | | | | | | | | |
| In sibling comparison analysis, model was adjusted for maternal age at delivery, parity, smoking during pregnancy, BMI in early pregnancy, maternal diabetic and hypertensive diseases, and child’s sex, calendar period of delivery, onset of labor, and mode of delivery. | | | | | | | | | |

| **Supplementary Table 8. Gestational age and risk of intellectual disability of different severity in non-malformed, term or post-term, appropriate-for-gestational-age children (Complete case analysis) (N=721094)** | | | | | | | | | |
| --- | --- | --- | --- | --- | --- | --- | --- | --- | --- |
| **Gestational age (weeks)** | **Population analysis** | | | | | | | | |
|  | **No. of children** | **Mild** | |  | **Moderate and Severe** | |  | **Other or unspecified** | |
|  |  | **No. of cases** | **HR (95%CI)** |  | **No. of cases** | **HR (95%CI)** |  | **No. of cases** | **HR (95%CI)** |
| Total | 721094 | 651 |  |  | 369 |  |  | 395 |  |
| 37-38 | 132997 | 134 | 1.30 (1.02-1.66) |  | 58 | 0.81 (0.58-1.14) |  | 90 | 1.37 (1.01-1.86) |
| 39 | 172855 | 161 | 1.26 (1.01-1.56) |  | 83 | 0.96 (0.73-1.28) |  | 96 | 1.21 (0.91-1.60) |
| 40 | 217163 | 160 | Ref |  | 108 | Ref |  | 99 | Ref |
| 41 | 140970 | 129 | 1.22 (0.96-1.54) |  | 71 | 0.98 (0.73-1.33) |  | 84 | 1.28 (0.96-1.71) |
| ≥42 | 57109 | 67 | 1.31 (0.96-1.77) |  | 49 | 1.49 (1.05-2.11) |  | 26 | 0.83 (0.53-1.29) |
|  | **Sibling comparison analysis** | | | | | | | | |
|  | **No. of children** | **Mild** | |  | **Moderate and Severe** | |  | **Other or unspecified** | |
|  |  | **No. of cases** | **HR (95%CI)** |  | **No. of cases** | **HR (95%CI)** |  | **No. of cases** | **HR (95%CI)** |
| Total | 245625 | 203 |  |  | 125 |  |  | 132 |  |
| 37-38 | 40987 | 37 | 0.93 (0.41-2.07) |  | 18 | 0.60 (0.19-1.89) |  | 25 | 3.45 (1.13-10.52) |
| 39 | 60831 | 49 | 0.68 (0.35-1.33) |  | 30 | 1.32 (0.57-3.04) |  | 35 | 1.57 (0.78-3.17) |
| 40 | 72671 | 55 | Ref |  | 38 | Ref |  | 35 | Ref |
| 41 | 50216 | 42 | 0.61 (0.27-1.40) |  | 22 | 0.61 (0.19-1.97) |  | 29 | 0.80 (0.33-1.94) |
| ≥42 | 20920 | 20 | 1.34 (0.51-3.49) |  | 17 | 1.53 (0.34-6.81) |  | 8 | 1.01 (0.20-5.19) |
| In population analysis, model was adjusted for maternal age at delivery, parity, educational level, country of birth, smoking during pregnancy, height, BMI in early pregnancy, maternal diabetic and hypertensive diseases, as well as child’s sex, calendar period of delivery, onset of labor, and mode of delivery. | | | | | | | | | |
| In sibling comparison analysis, model was adjusted for maternal age at delivery, parity, smoking during pregnancy, BMI in early pregnancy, maternal diabetic and hypertensive diseases, and child’s sex, calendar period of delivery, onset of labor, and mode of delivery. | | | | | | | | | |
